# Fast and simple decycling and dismantling of networks

**DOI:** 10.1038/srep37954

**Published:** 2016-11-29

**Authors:** Lenka Zdeborová, Pan Zhang, Hai-Jun Zhou

**Affiliations:** 1 Institut de Physique Thérique, CNRS, CEA and Université Paris-Saclay, Gif-sur-Yvette, France; 2 CAS Key Laboratory of Theoretical Physics, Institute of Theoretical Physics, Chinese Academy of Sciences, Beijing 100190, China

## Abstract

Decycling and dismantling of complex networks are underlying many important applications in network science. Recently these two closely related problems were tackled by several heuristic algorithms, simple and considerably sub-optimal, on the one hand, and involved and accurate message-passing ones that evaluate single-node marginal probabilities, on the other hand. In this paper we propose a simple and extremely fast algorithm, CoreHD, which recursively removes nodes of the highest degree from the 2-core of the network. CoreHD performs much better than all existing simple algorithms. When applied on real-world networks, it achieves equally good solutions as those obtained by the state-of-art iterative message-passing algorithms at greatly reduced computational cost, suggesting that CoreHD should be the algorithm of choice for many practical purposes.

In decycling of a network we aim to remove as few nodes as possible such that after the removal the remaining network contains no loop. In network dismantling we aim to find the smallest set of nodes such that after their removal the network is broken into connected components of sub-extensive size. These are two fundamental network-optimization problems with a wide range of applications, related to optimal vaccination and surveillance, information spreading, viral marketing, and identification of influential nodes. Considerable research efforts have been devoted to the network decycling and dismantling problems recently^1^,^2^,^3^,^4^,^5^,^6^,^7^,^8^.

Both the decycling and the dismantling problems belong to the class of NP-hard problems^6^,^9^, meaning that it is rather hopeless to look for algorithms to solve them exactly in polynomial time. However, finding the best possible approximate solutions for as large classes of networks as possible is an open and actively investigated direction. Recent theoretic and algorithmic progress on both these problems^1^,^2^,^3^,^5^,^6^ came from the fact that, on random sparse networks with degree distributions having a finite second moment, methods from physics of spin glasses provide accurate algorithms for both decycling and dismantling. These sparse random networks are locally tree-like and do not contain many short loops. On such networks the decycling is closely linked to dismantling and asymptotically almost the same set of nodes is needed to achieve both^5^,^6^,^10^. Even on real-world networks that typically contain many small loops, best dismantling is currently achieved by first finding a decycling solution and then re-inserting nodes that close short loops but do not increase too much the size of the largest component^5^,^6^.

Both the algorithms of refs 5 and 6 achieve performance that is extremely close to the theoretically optimal values computed on random networks. However, both these algorithms are global, they need to iterate certain equations on the whole network in order to select the suitable candidate nodes. Although they are both scalable and can be run on networks with many millions of nodes, they are not completely straightforward to understand and require some experience with spin glass theory. The close-to-optimal performance of these algorithms is theoretically justified only on random networks. Despite their good performance observed empirically on networks with many loops, there might still exist even better and analyzable strategies for real-world networks.

As usual in applied science, in many potential applications we are at first not even sure that optimal dismantling or optimal decycling is the best strategy to answer the question in hand (e.g., the problem of social influence maximization^11^,^12^,^13^,^14^). Therefore it is extremely important to have a really very simple and fast decycling and dismantling strategy that can provide an accurate assessment of whether this approach is at all interesting for a given practical problem. However, existing simple strategies, such as removing adaptively high degree nodes^15^,^16^, are very far from optimal performance and therefore not very useful. Recently the authors of ref. 4 claimed that a heuristics based on the so-called *collective influence* (CI) measure can be a perfect candidate for this purpose. This algorithm has attracted a lot of enthusiasm in the network science community. However, more systematic investigations performed in refs 5, 6 and 8 revealed that the CI algorithm is still considerably far from being optimal. The CI algorithm is also not particularly competitive in terms of computational time because a large neighborhood of a node needs to be considered in order to evaluate the CI measure.

In the present paper we introduce the CoreHD algorithm that is basically as simple and fast as the adaptive removal of high degree nodes, yet its performance is much closer to optimal than the CI algorithm or its extended versions, and comparably close as the best known message-passing methods^5^,^6^ while several orders of magnitude faster. It hence provides simple and tractable solutions for networks with many billions of nodes. The method is simply based on adaptive removal of highest-degree nodes from the 2-core of the network. Apart of its simplicity and speed the performance of the CoreHD algorithm is basically indistinguishable from the performance of the message-passing algorithms on random graphs with long-tailed degree distributions. On all real-world network instances we tested the result by CoreHD is within few nodes from the best one found by message-passing and on some instances we found that it is even slightly better. On top of all that, the simple structure of CoreHD might be amenable to rigorous analysis providing guarantees for loopy networks that are not accessible for the message-passing methods.

For all the above reasons we argue that in many applications of decycling and dismantling CoreHD should be the first choice. The simple algorithmic idea generalizes easily to the problem of destroying optimally the *k*-core of a network - one focuses on the current *k*-core and adaptively removes highest degree nodes.

## Results

In this section we evaluate the CoreHD algorithm for both random and real-world networks, by comparing the minimum fraction of nodes we need to remove in order to break the network into a forest or components with size smaller than 0.01 *N*. We compare to the Belief Propagation guided Decimation (BPD)^5^ and Collective Influence method (CI)^4^ (CI_4_ results are obtained using the original code of ref. 4).

First, we notice that on some simple examples, e.g. regular random graphs with degree 3, the CoreHD algorithm reaches the exact optimal decycling fraction *ρ* = 0.25. This matches the performance of a greedy method of ref. 17 that for this particular case is provably optimal.

**Figure 1:**
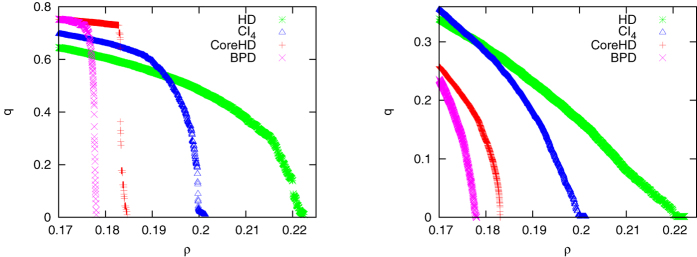
Fraction of nodes in the largest connected component (LCC) *(left)* and in the 2-core *(right)* as a function of fraction of nodes removed, for HD, CI_4_, CoreHD and BPD on an Erdös-Rényi random graph with number of nodes *N* = 5 × 10^4^, and average degree *c* = 3.5. In all four methods nodes are removed one by one.

In Fig. 1 we compare the performance of the above algorithms on an Erdös-Rényi random network with *N* = 50000 nodes and average degree *c* = 3.5. In the left panel we plot the fraction of nodes in the largest connected component (LCC, denoted *q*) as a function of the fraction of removed nodes, denoted *ρ*. We see that compared to HD and CI the CoreHD algorithm works the best by a large margin, breaking the network into small component with size smaller than 0.01 *N* after removing fraction of only 0.1846 of nodes. While CI and HD need to remove fraction 0.2014, and 0.2225 of nodes respectively. This is compared to the close-to-optimal performance of the iterative message passing BPD that needs to remove fraction 0.1780 of nodes, and to the theoretical prediction for the asymptotically optimal value 0.1753^1^,^2^,^3^,^6^,^7^.

We also see from the figure that the fraction of nodes in the LCC obtained by CoreHD encounters a first order transition at *ρ*_dec_ = 0.1831, this is because at this point (just at the beginning of the discontinuity) the remaining network becomes a forest. The greedy tree-breaking procedure then quickly breaks the forest into small components. While the other algorithms do not have this phenomenon, the size of the LCC goes to zero continuously. In the right panel of Fig. 1 we plot the fraction *q* of nodes in the 2-core as a function of *ρ*. We can see that for CoreHD, *q* reaches zero at *ρ* = 0.1831 indicating that the remaining network contains no loop, thus is a forest. While for other algorithms the 2-core remains extensive until the network is dismantled. On a larger ER random network with *N* = 10^6^, *c* = 3.5, the difference between the sizes of decycling and the dismantling sets the CoreHD algorithm finds is not distinguishable within the precision of 4 valid digits and is 0.1830 for both. Note that this result is (slightly) better than yet another approach suggested recently in the literature^8^ that achieves 0.1838 with an algorithm still considerably more involved than CoreHD.

Besides performing much better than CI, the CoreHD is also much faster: the 2-core of the network can be computed efficiently using a leaf-removal process with *O(N*) operations. After deleting a node, one only needs to update the 2-core, which requires on average *O*(1) operations in sparse networks, and is clearly much faster than updating the CI score. Actually, in sparse networks when the size of the 2-core is much smaller than the size of the network, CoreHD is even faster than the HD algorithm which removes one by one nodes from the whole network.

**Figure 2:**
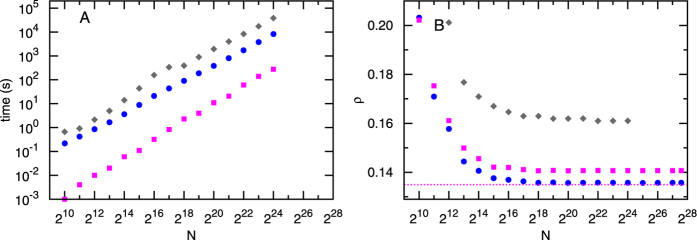
Performance of the CoreHD algorithm (magenta squares) and its comparison with the BPD algorithm (blue circles) and the CI algorithm (parameter 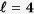
4, grey diamonds) on ER networks of average degree *c* = 3 and size *N*. (**A**) The relationship between the total running time *τ* and *N*. The simulation results are obtained on a relatively old desktop computer (Intel-6300, 1.86 GHz, 2 GB memory). (**B**) The relationship between the fraction *ρ* of removed nodes and *N*. The dotted horizontal line denotes the theoretically predicted minimum value.

The computational times for the CoreHG, CI and BPD algorithms as the system size grows are shown in Fig. 2 for ER network with mean degree *c* = 3. The BPD algorithm performs slightly better than the CoreHD algorithm but it is much slower. For example, for an ER network with *c* = 3 and *N* = 2 × 10^8^, the solution obtained by CoreHD has relative dismantling/decycling set size *ρ* ≈ 0.1407 (computing time is 64 minutes), which is only slightly larger than the value of *ρ* ≈ 0.1357 obtained by BPD (computing time is 23.5 hours^5^). We note that in these experiments, in each step of removal, BPD and CI_4_ remove 0.1% of nodes (e.g., 10000 nodes for *N* = 10^7^), while CoreHD removes only 1 node per step. Even this way the computational time of CoreHD is shorter than the time used for reading the network from the data file (edge-list format). We note that in the current implementation we use ordering of lists of *N* elements. This could be improved further, but we saw little point in doing so since the bottleneck was loading the graph, rather than the algorithm for decycling and dismantling.

**Figure 3:**
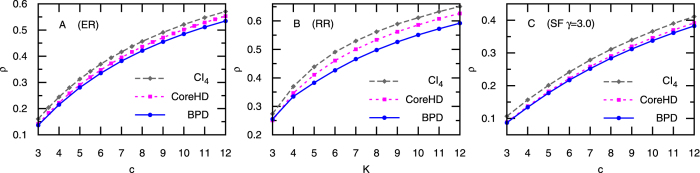
Fraction *ρ* of removed nodes for (**A**) Erdös-Rényi (ER) random networks of mean degree *c*, (**B**) Regular Random (RR) networks of degree *K*, and (**C**) Scale Free (SF) networks of average degree *c* and decay exponent *γ* = 3.0 generated as in ref. 20. Each data point obtained by CoreHD is the over 96 instances of size *N* = 10^5^. The results of CI_4_ and the results of BPD are from ref. 5. In BPD and CI_4_, at each iteration a fraction *f* of nodes are removed (with *f* = 0.01 for BPD and *f* = 0.001 for CI_4_, decreasing *f* does not improve the performance visibly), while in CoreHD nodes are removed one by one.

Figure 3 presents results for Erdös-Rényi random graphs, regular random graphs, and scale-free random networks of varying average degree. In all cases CoreHD works better than CI and worse than BPD, with the best performance obtained for scale-free networks. The good performance of CoreHD for the scale-free networks is of particular interest because almost all real-world networks have a heavy-tailed degree-distribution.

A set of experiments on real-world networks is presented in Table 1. We list the fraction of nodes we need to remove in order to remove all cycles, and in order to break the network into small components with size smaller than 0.01 *N*. For dismantling, in addition to Algorithm 6 we do a refinement by inserting back some deleted nodes that do not increase the largest component size beyond the 0.01 *N*. We can see that CoreHD works excellently for real-world network instances, giving decycling and dismantling sets very close to the state-of-art BPD and much smaller than CI. It is also surprising to see that in some networks e.g. RoadEU, IntNet1 and RoadTX, CoreHD even outperforms BPD slightly. Table 1 clearly demonstrates the time superiority of CoreHD for real-world networks as compared with both CI and BPD.

Note that in recent work^6^ authors stressed that in random graphs there are many dismantling sets of size close to the optimum, and a given node can be included in some and not included in others. Such an observation is quite common to many optimization problems on networks where typically the ground state of a given optimization problem is degenerate and different low-energy configurations may disagree on a considerable number of nodes. The CoreHD algorithm is also able to find different dismantling sets since nodes to be removed are chosen among the typically numerous ones in the core having the largest degree, by running the algorithm with different random number initialization for the ER network of average degree 3.5 we typically observed that two different dismantling sets obtained by the CoreHD algorithm agree in 74% of nodes. From this observation we conclude, in agreement with^6^, that the concept of dismantling set is a result of highly correlated choice and not addition of a set of particularly important nodes (sometimes refer to as influential nodes or super-spreaders^4^.

## Discussion

We have presented that iteratively removing nodes having the highest degree from the 2-core of a network gives an ultra-fast while very efficient algorithm for decycling and dismantling of networks. Our algorithm is so fast that its running time is shorter than the time of reading the network file.

It is still surprising to us that such a simple algorithm could work much better than more sophisticated algorithms: We have tried running CI (see SI), adjacency matrix centrality on the 2-core of the network, and HD on 3-core of the network, they are all slower but perform no better than CoreHD. Our experiments also show that CoreHD outperforms centrality measures using left and right eigenvector of the non-backtracking matrix^18^, an idea that originally inspired us to propose the CoreHD algorithm. More detailed understanding of why this is the best performing strategy is let for future work.

On the real-world networks which typically have many short loops and motifs, decycling is quite different from dismantling. A natural idea to generalize our CoreHD would be consider a factor graph treating short loops and motifs as factors, then do CoreHD on the 2-core of the factor graph.

Finally, CoreHD can be generalized naturally to removal of the *k*-core, again running the adaptive HD heuristics on the *k*-core or the current graph. Comparison of this strategy to existing algorithms^2^,^19^ is in progress.

## Methods

We now describe CoreHD as an extremely fast algorithm for decycling and dismantling of huge complex networks with close-to-optimal outcomes. Let us begin with some motivating discussions.

**Figure 4:**
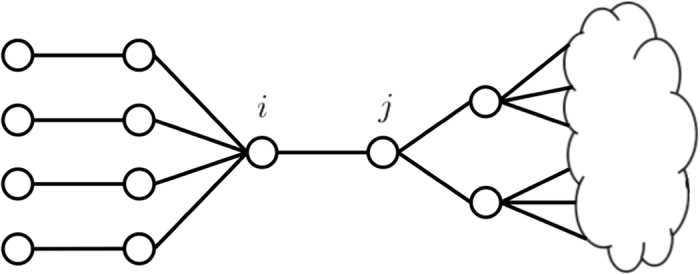
Illustration of a network with dangling trees. Each circle denotes a node in the network, each line connecting circles denotes an edge, and the cloud represents the other part (nodes and edges) of the network.

Perhaps the simplest algorithms one can propose for decycling and dismantling is adaptive removal of highest-degree nodes. We call this method HD, it is indeed extremely fast, but empirically does not perform very well. One reason why HD does not work well is that some nodes of large degree, such as node *i* in Fig. 4, do not belong to any loop, and hence do not have to be removed for decycling. Due to the property that trees can always be dismantled by a vanishing fraction of nodes^10^, nodes such as *i* of Fig. 4 are also not useful for dismantling. Note that the CI method of ref. 4 shares this problem, see the SI for the argument on why our algorithm outperforms CI.

The above observation motivates a very natural idea that dismantling and decycling algorithms should always focus only on the 2-core of the network. The 2-core is a sub-network that is obtained after adaptive removal of all leaves (nodes with only a single attached edge). The simplest and fastest strategy is then to remove the highest-degree nodes from the remaining 2-core. To our surprise this simple idea provides much better performance than other comparably simple approaches existing in the literature. We call the resulting algorithm CoreHD, it is detailed in Algorithm 1.


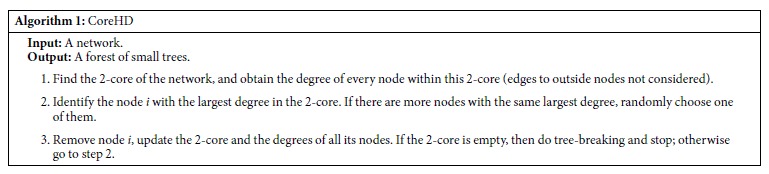


For the decycling problem, CoreHD simply removes highest-degrees nodes from the 2-core in an adaptive way (updating node degree as the 2-core shrinks), until the remaining network becomes a forest. For dismantling, after decycling, CoreHD also breaks the trees into small components, see SI that follows tree-breaking strategy from refs 5 and 6. In case the original network has many small loops, a refined dismantling set is obtained after a reinsertion of nodes that do not increase (much) the size of the largest component, again as proposed recently in refs 5 and 6. For details on implementation of the reinsertion algorithm we refer to the SI.

## Additional Information

**How to cite this article**: Zdeborová, L. *et al.* Fast and simple decycling and dismantling of networks. *Sci. Rep.* **6**, 37954; doi: 10.1038/srep37954 (2016).

**Publisher's note:** Springer Nature remains neutral with regard to jurisdictional claims in published maps and institutional affiliations.

## Supplementary Material

Supplementary Information

## Figures and Tables

**Table 1:** Comparative results of the CoreHD method with CI and the BPD algorithm on a set of real-world network instances.

Network	*N*	*M*	decycling		dismantling			Time for dismantling		
			CoreHD	BPD	CI	CoreHD	BPD	CI	CoreHD	BPD
RoadEU^21^	1177	1417	**90**	91	209	**148**	152	0.18	<0.001	0.1
PPI^22^	2361	6646	365	362	424	357	350	0.91	<0.001	2.09
Grid^23^	4941	6594	519	512	476	327	320	1.00	<0.001	0.66
IntNet1^24^	6474	12572	217	215	198	**156**	161	5.19	<0.001	11.32
Authors^25^	23133	93439	**8311**	8317	3588	2600	2583	87.55	0.09	40.04
Citation^24^	34546	420877	15489	15390	14518	13523	13454	4166	0.2	383.91
P2P^26^	62586	147892	9557	9285	10726	9561	9292	520.59	0.21	50.24
Friend^27^	196591	950327	38911	38831	32340	27148	26696	5361	1.37	588.19
Email^25^	265214	364481	1189	1186	21465	1070	1064	6678	0.39	151.57
WebPage^28^	875713	4322051	**208509**	208641	106750	51603	50878	2275	9.67	2532
RoadTX^28^	1379917	1921660	243969	239885	133763	**20289**	20676	273.69	4.07	421.15
IntNet2^24^	1696415	11095298	229034	228720	144160	73601	73229	19715	35.84	4243

*N* and *M* are the number of nodes and links of each network, respectively. The number of nodes deleted by CI, CoreHD, and BPD are listed in the 4th, 5th, and 6th column. The CI and BPD results are from ref. 5. The time (seconds) for dismantling is the running time of algorithms, i.e. with time for reading network from the data file excluded.
