# Supplementary material for: Fast and simple decycling and dismantling of networks
Source: Sci Rep. 2016 Nov 29;6:37954. doi: 10.1038/srep37954 (PMC5126572; doi:10.1038/srep37954)
Supplement: Supplementary Information [file srep37954-s1.pdf]

Supporting Information for: Fast and simple decycling and dismantling of networks

Lenka Zdeborová
Institut de Physique Thérique, CNRS, CEA and Université Paris-Saclay, Gif-sur-Yvette, France

Pan Zhang and Hai-Jun Zhou
CAS Key Laboratory of Theoretical Physics, Institute of Theoretical Physics, Chinese Academy of Sciences, Beijing 100190, China

(Dated: October 29, 2016)

GREEDY TREE BREAKING AND REFINEMENT BY INSERTION

Optimally breaking a forest into small components can be solved in polynomial time [1]. Empirically a greedy tree-breaking procedure works very well. In such a greedy dynamics we iteratively find and remove the node which leads to the largest drop in the size of the largest connected component.

In more details, the largest component caused by removal of each node in a tree can be computed iteratively (see, e.g. [2, 3]). Starting from a leaf, each node sends a message to each of its neighbors, reporting the largest component caused by removing the edge between them. After the messages arrive at the root of the tree, we can then easily identify the node such that its removal decreases maximally the component size.

For the refinement, we also use a simple greedy strategy to insert back some of the removed nodes [2, 3]. In each step of re-insertion, we calculate the increase of the component size after the insertion of a node, and then identify the node which gives the smallest increase.

DANGLING-TREE PROBLEM OF THE CI INDEX

The collective influence index was proposed in [4] as a measure of node's importance in influence spreading. At a given level ℓ the CI index of a node i is defined as

$$\mathrm{CI}_\ell(i) = (d_i - 1) \sum_{j \in \partial_i^\ell} (d_j - 1) \ , \tag{1}$$

where d_i is the degree of node i in the remaining network, and ∂_i^ℓ denotes the set of nodes that are at distance ℓ from node i. In the CI algorithm, a small fraction f (e.g., $f = 0.001$) of nodes with the highest CI values are removed from the network and then the CI indices of the remaining nodes are updated. The authors of [4] claimed that the $\mathrm{CI}_\ell(i)$ approximates the eigenvector of the non-backtracking operator [5].

However we can see immediately that CI has a drawback which does not reflect the functioning of the non-bactracking operator. We illustrate this in an example network shown in Fig. **??**. Without loss of generality let us consider $\ell = 2$, then it is easy to see that the node i of this figure has $\mathrm{CI}_2(i) > 0$ and in some cases can be larger than the CI indices of the other nodes. So the CI algorithm may say node i is more important to remove first, as its removal decreases mostly the eigenvalue of the non-backtracking matrix. After a moment of thought we see that this conclusion is not correct, as removing node i does not change the eigenvalue of the non-backtracking matrix at all, because the eigenvalue of the non-backtracking matrix is the same as the 2-core of the network, while node i does not belong to the 2-core of the network.

COMPARING CORE-HD AND CORE-CI

Since performing node deletion on the network 2-core is the key of CoreHD's good performance, it is natural to expect that the CI algorithm can also be improved by adding the 2-core reduction process. To confirm this, we implement an extended CI algorithm (named as CoreCI) as follows. At each elementary node removal step, (1) the 2-core of the remaining network is obtained by cutting leaves recursively as in CoreHD, and then (2) the CI index of each node in the 2-core is computed by considering only nodes and links within this 2-core, and finally (3) a node with the highest CI index is deleted from the 2-core. Similar to CoreHD and BPD, after a forest is produced by CoreCI,

TABLE I. Comparing the dismantling performance of CoreHD and CoreCI on ER, RR, and SF random networks. Each data point is the mean and standard deviation of ρ (the fraction of deleted nodes) over 96 dismantling solutions obtained by CoreHD or CoreCI on 96 independent network instances of size $N = 10^5$ and mean degree c (ER and SF) or degree K (RR). The ball radius of CoreCI is fixed to $\ell = 4$. The SF network instances are generated by the static method [6].

ER			RR			SF ($\gamma = 3.0$)		
c	CoreHD	CoreCI	K	CoreHD	CoreCI	c	CoreHD	CoreCI
3.0	0.1413(3)	0.1427(3)	3	0.25043(3)	0.2539(2)	3.0	0.0886(3)	0.0893(3)
4.0	0.2226(4)	0.2249(4)	4	0.3464(2)	0.3564(3)	4.0	0.1373(4)	0.1383(4)
5.0	0.2908(4)	0.2937(4)	5	0.4110(2)	0.4239(3)	5.0	0.1820(5)	0.1833(5)
6.0	0.3476(4)	0.3509(4)	6	0.4605(3)	0.4733(3)	6.0	0.2222(5)	0.2237(5)
7.0	0.3954(4)	0.3990(4)	7	0.5004(3)	0.5128(3)	7.0	0.2582(5)	0.2560(5)
8.0	0.4361(4)	0.4400(5)	8	0.5335(3)	0.5455(3)	8.0	0.2906(6)	0.2925(6)
9.0	0.4712(4)	0.4752(5)	9	0.5617(3)	0.5733(3)	9.0	0.3196(5)	0.3217(5)
10.0	0.5018(4)	0.5060(4)	10	0.5861(3)	0.5974(4)	10.0	0.3460(6)	0.3481(6)
11.0	0.5288(4)	0.5330(4)	11	0.6075(3)	0.6182(4)	11.0	0.3699(6)	0.3723(6)
12.0	0.5527(4)	0.5571(4)	12	0.6264(3)	0.6367(3)	12.0	0.3918(6)	0.3943(6)

we then perform a greedy tree-breaking process if necessary and then re-insert some nodes back to the network as long as the size of the largest connected component is still below the threshold value of (say) $0.01N$.

We indeed observe that CoreCI performs considerably better than the original CI algorithm. However it does not outperform CoreHD. We list in Table I the comparative results of CoreHD versus CoreCI on ER, RR, and SF random networks. Notice that the fractions ρ of deleted nodes by CoreHD and CoreCI are very close to each other, with CoreHD performs slightly better. These results clearly demonstrate that the CI index is not a better indicator of node importance than the degree in the 2-core. Because repeatedly computing the CI indices within the 2-core is still very time-consuming, we recommend CoreHD rather than CoreCI as an efficient heuristic for practical applications.

[1] S. Janson and A. Thomason. Dismantling sparse random graphs. *Combinatorics, Probability and Computing*, 17(02):259–264, 2008.

[2] Salomon Mugisha and Hai-Jun Zhou. Identifying optimal targets of network attack by belief propagation. *Phys. Rev. E*, 94:012305, Jul 2016.

[3] Alfredo Braunstein, Luca DallAsta, Guilhem Semerjian, and Lenka Zdeborov. Network dismantling. *Proceedings of the National Academy of Sciences*, 2016.

[4] F. Morone and H. A. Makse. Influence maximization in complex networks through optimal percolation. *Nature*, 524:65–68, 2015.

[5] F. Krzakala, C. Moore, E. Mossel, J. Neeman, A. Sly, L. Zdeborová, and P. Zhang. Spectral redemption in clustering sparse networks. *Proc. Natl. Acad. Sci. USA*, 110(52):20935–20940, 2013.

[6] K.-I. Goh, B. Kahng, and D. Kim. Universal behavior of load distribution in scale-free networks. *Phys. Rev. Lett.*, 87:278701, 2001.
